# Reactive Neurogenesis and Down-Regulation of the Potassium-Chloride Cotransporter KCC2 in the Cochlear Nuclei after Cochlear Deafferentation

**DOI:** 10.3389/fphar.2016.00281

**Published:** 2016-08-31

**Authors:** Brahim Tighilet, Sophie Dutheil, Marina I. Siponen, Arnaud J. Noreña

**Affiliations:** ^1^Laboratoire de Neurosciences Intégratives et Adaptatives, UMR 7260 – Comportement, Cerveau, Cognition (Behavior, Brain, and Cognition) – Aix-Marseille Université – Centre National de la Recherche ScientifiqueMarseille, France; ^2^Department of Psychiatry, School of Medicine, Yale University, New HavenCT, USA

**Keywords:** neurogenesis, KCC2 transporter, hearing loss, sensorineural, tinnitus and hyperacousis, homeostatic plasticity

## Abstract

While many studies have been devoted to investigating the homeostatic plasticity triggered by cochlear hearing loss, the cellular and molecular mechanisms involved in these central changes remain elusive. In the present study, we investigated the possibility of reactive neurogenesis after unilateral cochlear nerve section in the cochlear nucleus (CN) of cats. We found a strong cell proliferation in all the CN sub-divisions ipsilateral to the lesion. Most of the newly generated cells survive up to 1 month after cochlear deafferentation in all cochlear nuclei (except the dorsal CN) and give rise to a variety of cell types, i.e., microglial cells, astrocytes, and neurons. Interestingly, many of the newborn neurons had an inhibitory (GABAergic) phenotype. This result is intriguing since sensory deafferentation is usually accompanied by enhanced excitation, consistent with a reduction in central inhibition. The membrane potential effect of GABA depends, however, on the intra-cellular chloride concentration, which is maintained at low levels in adults by the potassium chloride co-transporter KCC2. The KCC2 density on the plasma membrane of neurons was then assessed after cochlear deafferentation in the cochlear nuclei ipsilateral and contralateral to the lesion. Cochlear deafferentation is accompanied by a strong down-regulation of KCC2 ipsilateral to the lesion at 3 and 30 days post-lesion. This study suggests that reactive neurogenesis and down-regulation of KCC2 is part of the vast repertoire involved in homeostatic plasticity triggered by hearing loss. These central changes may also play a role in the generation of tinnitus and hyperacusis.

## Introduction

It has been suggested that homeostatic regulation applies to the averaged neural activity as many neural processes, such as long-term potentiation and depression, can produce unstable activity, i.e., runaway activity or no activity at all, respectively (Davis and Goodman, 1998; Turrigiano and Nelson, 1998; Turrigiano et al., 1998). Changes compatible with homeostatic regulation of neural activity have been reported throughout the central nervous system, including the sensory systems (Turrigiano, 1999; Turrigiano and Nelson, 2004; Watt and Desai, 2010), and after various manipulations or lesions. In the auditory modality, an enriched acoustic environment provided for a few weeks has been shown to reduce cortical excitability within the frequency region stimulated by the acoustic environment (Noreña et al., 2006; Pienkowski et al., 2011, 2013). On the other hand, moderate to profound hearing loss is accompanied by an increase of spontaneous and stimulus-induced activity at many levels of the central auditory system, from the cochlear nucleus (CN) up to the auditory cortex (Kaltenbach et al., 2000; Noreña et al., 2003; Sumner et al., 2005; Mulders and Robertson, 2009; Kalappa et al., 2014).

The repertoire of cellular and molecular mechanisms involved in homeostatic plasticity is considerable (Burrone and Murthy, 2003; Turrigiano and Nelson, 2004; Davis, 2006; Turrigiano, 2008; Watt and Desai, 2010). Among many other mechanisms, it has been shown that the number of post-synaptic receptors and voltage-gated channels at the level of the axon initial segment are precisely regulated (Turrigiano, 1999; Grubb and Burrone, 2010; Kuba et al., 2010). It is also well known that the balance between excitation and inhibition is finely adjusted (Rutherford et al., 1998; Kilman et al., 2002). Moreover, reactive neurogenesis has been demonstrated in the vestibular nuclei after a unilateral vestibular nerve section. Interestingly, many of the newborn neurons following the deafferentation were of GABAergic phenotype (Tighilet et al., 2007; Dutheil et al., 2013). At first sight, this result is surprising as sensory deafferentation is usually followed by neural hyperexcitability (Noreña et al., 2003; Sumner et al., 2005; Mulders and Robertson, 2009; Kalappa et al., 2014), consistent with a reduction of inhibitory neurotransmission (Suneja et al., 1998; Milbrandt et al., 2000; Argence et al., 2006). Importantly, however, the polarity of GABA action on membrane potential is regulated in adults by KCC2 co-transporters which maintain intra-cellular concentration of chloride ion at low levels (Payne et al., 2003; De Koninck, 2007). As a consequence, a down-regulation of KCC2 after various manipulations have been shown to make the polarity of GABA depolarizing (Coull et al., 2003; Boulenguez et al., 2010).

By definition, homeostatic plasticity in the nervous system is considered to be adaptive. In some cases, however, and for reasons that are still unclear, homeostatic plasticity may be accompanied by collateral effects that can lead to neurological diseases (Schaette and Kempter, 2006; Wondolowski and Dickman, 2013; Kotas and Medzhitov, 2015). In particular, a down-regulation of KCC2 may play a key role in the pathogenesis of epilepsy, neuropathic pain, and spasticity (Cohen et al., 2002; Coull et al., 2003; De Koninck, 2007; Doyon et al., 2013; Kaila et al., 2014). In this regard, investigating how the central nervous system maintains a stable averaged activity around a set point level is a fundamental question in neuroscience and also a pre-requisite to understand some pathological conditions of the central nervous system.

The aim of the present study was to further explore the cellular and molecular mechanisms of homeostatic plastic after unilateral cochlear nerve section in the first relay of the central auditory system, the CN. Tinnitus, an auditory perception not induced by an external sound, and hyperacusis, a hypersensitivity to sound, have been suggested to be a by-product of homeostatic plasticity triggered by hearing loss (Schaette and Kempter, 2006; Noreña, 2011; Noreña and Farley, 2012). In this context, a secondary goal of the present study was to gain further insight into the mechanisms of tinnitus and hyperacusis. Neural proliferation and the phenotype of the newborn neurons were studied in the different sub-divisions of the CN ipsilateral and contralateral to the lesion. Moreover, in order to investigate whether the sensory deafferentation reverses the hyperpolarizing effect of GABA on the membrane potential, the density of the co-transporter KCC2 on the neuronal plasma membrane was also assessed.

## Materials and Methods

### Ethics Statement

All experiments were carried out in strict accordance with the National Institutes of Health Guide for Care and Use of Laboratory Animals (NIH Publication n° 80–23) revised 1996 for the UK Animals (Scientific Procedures) Act 1986 and associated guidelines, or the Policy on Ethics approved by the Society for Neuroscience in November 1989, and amended in November 1993. Male European cats used in the experiments were supplied by ISOQUIMEN (Barcelona, SPAIN) and were housed in our animal housing facility (Fédération 3C, Centre Saint-Charles, Aix-Marseille University) under the veterinary and National Ethical Committee supervision (French Agriculture Ministry Authorization: B13-055-25). Every attempt was made to minimize both the number and the suffering of animals used in this experiment. We selected only the most important post-UCN time delay in light of the findings of our previous studies and in order to limit the number of cats used. Animals were housed in a large confined space with normal diurnal light variations and free access to water and food.

### Surgery

Adult male cats weighing between 4 and 5 kg were anesthetized with ketamine (20 mg/kg, i.m.; Rhône-Poulenc, Mérieux, France), received an analgesic (Tolfedine, 0.5 ml, i.m.; Vetoquinol, Lure, France) and were kept at physiological body temperature using a blanket. The cochlear nerve was sectioned on the left-side at the post-ganglion level after mastoidectomy, partial destruction of the bony labyrinth, and surgical exposure of the internal auditory canal. Animals were maintained under antibiotics for 7 days and analgesics for 3 days.

### Implantation and Use of Osmotic Minipumps for Drug Infusion in the Fourth Ventricle

For the implantation and use of osmotic minipumps containing cytosine-ß-D arabinofuranoside (AraC, S-phase-specific antimitotic drug, Sigma–Aldrich, Saint-Quentin Fallavier, France), Muscimol (GABA_A_ receptor agonist, Sigma–Aldrich, Saint-Quentin Fallavier, France) or 0.9% sodium chloride (NaCl), a stainless steel cannula was implanted under anesthesia into the fourth ventricle of the brain and connected to a subcutaneous minipump (Alzet, Alza Corporation, Palo Alto, CA, USA; flow rate 2.5 μl/h for 30 days). A midline incision was made through the skin and musculature in the back of the neck, and a cannula connected to plastic tubing was inserted between the dorsal wall of the brainstem and the ventral face of the cerebellum and then cemented with dental cement to the skull. The air in the system was removed by filling up with saline, muscimol or AraC, after which the tubing was connected to an osmotic minipump, and the skin was incised. Muscimol was diluted to the required concentration using artificial cerebrospinal fluid (124 mM NaCl, 5 mM KCl, 1.2 mM KH2PO4, and 1.3 mM MgSO4) and tested for pH and adjusted to a pH 7.0, if necessary. As detailed in the study of Gliddon et al. (2005), we chose concentrations that provided an effect on vestibular neurogenesis without adverse side effects on animals (Dutheil et al., 2013). AraC was diluted in a NaCl solution at 0.13 mM, this concentration was demonstrated to inhibit totally the mitotic activity in the deafferented vestibular nuclei without adverse side effects on animals (Dutheil et al., 2009). Cats were infused continuously into the cerebrospinal fluid of the fourth ventricle during 3 or 30 days to study the consequences of the drugs on cell proliferation, survival, and differentiation.

### Study Design

**FIGURE 1:**
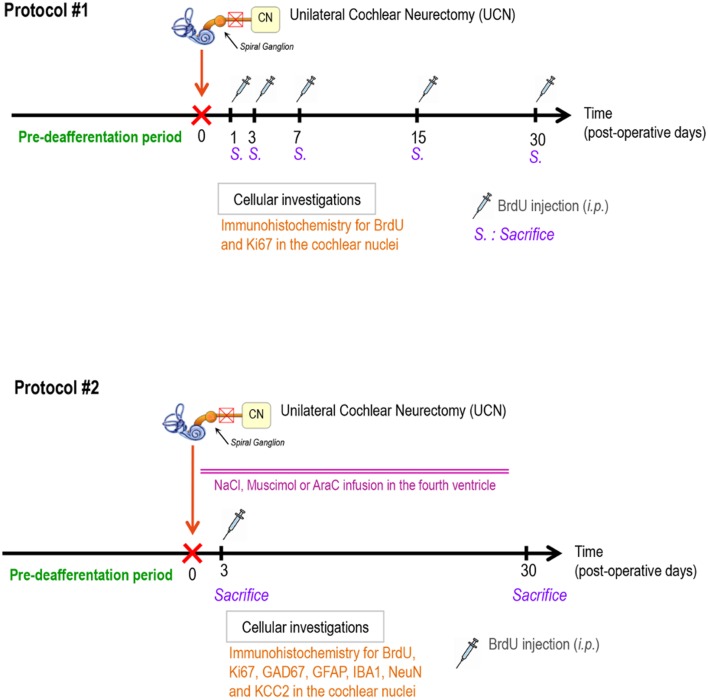
**Study designs.** The first protocol (#1) was elaborated using BrdU and Ki67 as cell proliferation markers to study the time-dependent effect of a UCN on reactive cell proliferation in cochlear nuclei. Protocol number 2 (#2) was designed using a continuous infusion of NaCl, AraC, or Muscimol in the fourth ventricle to examine alterations in newborn cell survival (BrdU marker), astrogenesis (GFAP marker), GABA cells (GAD67 marker), neuronal cells (NeuN marker), and microglial cells (IBA1 marker) in the cochlear nuclei. BrdU, 5-bromo-2′deoxyuridine; i.p., intraperitoneal injection; GAD67, glutamic acid decarboxylase; GFAP, glial fibrillary acidic protein; CN, cochlear nuclei; *n* = 4 animals per group.

A total of 24 cats were used to determine the time course of cell proliferation in the cochlear nuclei (first experimental protocol: **Figure 1**). Among these cats, 20 were subjected to a unilateral cochlear neurectomy (UCN). They were injected intraperitoneally (i.p.) with 5-bromo-2′deoxyuridine (BrdU: 200 mg/kg) 3 h before they were killed and perfused. BrdU is a thymidine analog that can be incorporated into DNA during the S phase of the cell cycle. Five post-UCN survival periods were used: 1, 3, 7, 15, and 30 days. Each of these survival groups was composed of four cats. The survival periods were selected based on our previous investigations, which had showed a high number of BrdU-immunoreactive nuclei in the deafferented vestibular nuclei with a peak at 3 days after unilateral vestibular neurectomy and a decrease at 30 days (Tighilet et al., 2007). A control group was made up of four sham-operated cats. This group was subjected to the same anesthetic procedure and surgical approach on the cochlear nerve but without sectioning the nerve. They were injected with the same amount and route of administration of BrdU 3 days after sham surgery and killed 3 h later.

To study the survival and the differentiation of the proliferating cells and to determine the effects of NaCl, muscimol or AraC infusion after UCN on the different steps of reactive neurogenesis (proliferation, survival, and differentiation) at the cellular level (second experimental protocol, **Figure 1**), we studied six groups of male adult cats: (i) UCN-NaCl groups, animals underwent UCN with continuous NaCl infusion, then received an, i.p., BrdU injection (200 mg/kg, i.p.) and were killed at either D3 when cell proliferation reached a peak (group 1, *n* = 4) or D30 to study the survival and the differentiation of the proliferating cells (group 2, *n* = 4); (ii) UCN-Muscimol groups, animals underwent UCN with continuous Muscimol infusion, then received an, i.p., BrdU injection and were killed at D3 (group 3, *n* = 4) or D30 (group 4, *n* = 4); (iii) UCN-AraC groups, animals underwent UCN with continuous AraC infusion, received an, i.p., BrdU injection and were killed at D3 (group 5, *n* = 4) or D30 (group 6, *n* = 4). The two post-deafferentation survival periods (D3 and D30) were selected on the basis of our anterior data (Tighilet et al., 2007).

### Cellular Investigations

#### Tissue Preparation

BrdU (10 mg/ml, Sigma, Saint Quentin Fallavier, France) was dissolved in a solution of sodium chloride (NaCl) 0.9% heated to 56°C and injected into animals (200 mg/kg). It has been shown in adult rat dentate gyrus that a single dose of BrdU 100, 50, or 25 mg/kg (body weight, i.p.) labeled 60, 45, and 8% of S-phase cells, respectively (Cameron and McKay, 2001). At 300 mg/kg, BrdU labeled most S-phase cells and had no physiological side effects. So, in line with the conclusions of Taupin (2007), we considered 200 mg/kg as a saturating concentration of BrdU for studying adult neurogenesis. BrdU doses were not likely to generate side effects, but were sufficient to mark the cells in S-phase synthesizing DNA. Before BrdU administration, the cats of each group were deeply anesthetized with ketamine dihydrochloride (20 mg/kg, i.m., Merial, Lyon, France) and killed by 0.9% NaCl (1L per animal) then paraformaldehyde 4% (2L per animal) transcardiac perfusion either 3 h or 27 days later according to their experimental group. After removal from the skull, brains were cut into several blocks containing the cochlear nuclei. The blocks were rapidly frozen with dry ice and stored at -80°C. Coronal sections (40-μm-thick) were cut in a cryostat (Leica, Rueil-Malmaison, France) for immunochemistry.

#### Immunochemistry

Immunochemical labeling was performed according to previously validated protocols (Tighilet et al., 2007). Ki67 marker was used in addition to BrdU to confirm that BrdU had been incorporated into mitotic cells and did not correspond to dying cells or a DNA repair mechanism (Dutheil et al., 2009). For BrdU immunostaining, free-floating sections were first rinsed in 0.1 M PBS and incubated with 2N HCl and 0.5% Triton-X100 in PBS (30 min, 37°C) for DNA hydrolysis. Then sections were rinsed in 0.1 M sodium tetraborate buffer, pH 8.5 before overnight incubation with the primary antibody at 4°C, followed by incubation with the secondary antibody for 1.5 h at room temperature, and visualized using horseradish peroxidase avidin-D (Vector). GFAP and GAD67 immunoreactivity assays were also performed (Tighilet et al., 2007). After several rinses, sections were mounted on gelatin-coated slides, dehydrated, and cover-slipped in Depex mounting medium for peroxidase staining. The differentiation of the newly generated cells was analyzed in the group of cats injected with BrdU 3 days after UCN and killed after 1 month. We used double immunofluorescent stained sections incubated with BrdU and one of four antibodies: NeuN, a post-mitotic neuronal nuclei marker expressed in most neurons; the glial fibrillary acidic protein (GFAP), a specific type of intermediate filament protein used as astrocyte marker; IBA1, a ionized calcium binding adapter molecule 1, specific to microglia and macrophages but not cross-reactive with neurons and astrocytes; and GAD 67, the enzyme that catalyzes the decarboxylation of glutamate to GABA and expressed in GABAergic neurons. Each antibody was processed sequentially, the differentiation marker detection first and then the BrdU labeling. For fluorescent labeling, sections were incubated with a secondary antibody cover-slipped in Mowiol. Differentiation of the newly generated cells was analyzed with double-labeling analysis performed using confocal imaging with a Zeiss LM 710 NLO laser scanning microscope equipped with a 63x/1.32 NA oil immersion lens. The fields of view were then examined by confocal microscopy, and 1-μm-step Z series were obtained.

Immunochemical labeling for KCC2 was performed according to previously validated protocols (Boulenguez et al., 2010; Bos et al., 2013). We incubated sections overnight at 22°C in a mixture of affinity-purified rabbit KCC2-specific polyclonal antibody (1:200; Millipore). We then revealed the labeling with a mixture of donkey Cy3-conjugated rabbit-specific antibody (1:500, Jackson Immunoresearch), and mounted coverslips with a gelatinous aqueous medium. We analyzed the patterns of immunolabeling by means of a laser scanning confocal microscope (Zeiss LSM 710 META) at high magnification (Plan Apochromat 63x 1.4 (N.A) oil immersion objective).

#### Negative Control and Antibody Specificity

We performed control experiments consisting in omitting successively one of the primary antibodies resulting in a complete absence of cross-reactivity.

#### Cell Counts and Statistical Analysis

Cell counts were performed according to a previously validated protocol (Dutheil et al., 2009, 2011). Great care was taken not to count blood cells as BrdU^+^ cells. The cochlear nuclei were identified according to Berman’s stereotaxic atlas (Berman, 1968). BrdU^+^, Ki67^+^, GFAP^+^, and GAD67^+^ were quantified for each subdivision of the CN, namely dorsal, posteroventral, anteroventral, and cochlear granular cell layer (DCN, PVCN, AVCN, and CGL, respectively). BrdU^+^ cells, Ki67^+^cells, GFAP^+^ cells, and GAD67^+^ neurons were analyzed in each CN on both sides (left/right in sham-operated cats and ipsilateral/contralateral to the lesion in UCN-lesioned cats). While it is usually straightforward to distinguish large- and middle-sized neurons from glial cells, the distinction between small neurons and large glial cells can be challenging. Thus, the following criteria were used as characteristic to distinguish small GAD 67^+^ neurons from GFAP^+^ glial cells at D30: a centrally located nucleolus, a distinctive nucleus, visible cytoplasm, presence of dendritic processes, and larger cell body size. Hence, glial cells were identified by sparse cytoplasm and smaller cell body size (Christensen et al., 2007). The cell count was done with a Nikon microscope (Eclipse 80 i) equipped with a motorized X-Y-Z sensitive stage and a video camera connected to a computerized image analysis system (Mercator; Explora Nova, La Rochelle, France). The total number of immunolabeled cells was estimated using the optical fractionator method (West et al., 1991). BrdU^+^, Ki67^+^, GFAP^+^, NeuN^+^, IBA1^+^, and GAD67^+^ were counted and sampled according to the so-called fractionator principles, that is, a combination of the optical dissector, a three-dimensional probe used for counting, and fractionator sampling, a scheme involving the probing of a known fraction of the tissue (West, 1993). This cell counting method has been described and validated in previous publications (Dutheil et al., 2009, 2011). Accordingly, the statistical analyses were evaluated by ANOVA to test the effects of the group (UCN-NaCl, -Musimol or -AraC), the CN sub-division (DCN, PVCN, AVCN, and CGL), and the post-operative time on Ki 67^+^, BrdU^+^, GFAP^+^, KCC2^+^, and GAD67^+^ cells and to determine whether there were any interactions between these variables. ANOVA was followed by *post hoc* analysis with the Scheffé test. Differences between sub-divisions of CN were considered statistically significant when *p* < 0.05 (StatView II, SAS software Inc., Cary, NC, USA).

### Quantification of KCC2 Immunohistological Labeling

Double fluorescent labelings were captured using frame-channel mode to avoid any cross talk between the channels. Each optical section resulted from two scanning averages. Excitation of the fluorochromes was performed with an argon ion laser set at 488 nm, and a helium/neon laser set at 575 nm. At high magnification, we only scanned the ventral CN (PVCN and AVCN regions), which exhibited reactive neurogenesis. We digitized stacks of 1 μm-thick optical sections.

A custom program written in Matlab^®^ (The Mathworks, Inc.) was developed to analyze fluorescence at the plasma membrane of neurons. The background or non-specific immunofluorescence, was assessed by calculating the average fluorescence in a visually selected area devoid of neurons or any other stained structures. From this region, we then derived a threshold equal to the average immunofluorescence plus three times the standard deviation. All data were then subtracted from this threshold value and only positive values were conserved for further analysis. A region of interest was then drawn around the neuronal plasma membrane of each cell body. The program calculated the average fluorescence within the region of interest over data that were 20% above the maximum values. This thresholding insured that all pixels taken for calculating the average was part of the plasma membrane and that the same criterion was used for all slices in all conditions (otherwise, the averaged fluorescence can depend on how large the region of interest was manually drawn around the plasma membrane).

Also, to avoid any biases at the stage of extracting the averaged fluorescence level in each condition, the experimenter in charge of the analysis (M.I.S.) ignored the conditions corresponding to the slices. We used the non-parametric Mann–Whitney test to compare control and lesioned animals, and the lesioned and intact side at both 3 and 30 days post-lesion in lesioned animals. Differences were considered statistically significant at *p* < 0.05.

## Results

### Time Course of BrdU and Ki67 Immunoreactivity in Cochlear Nuclei

**FIGURE 2:**
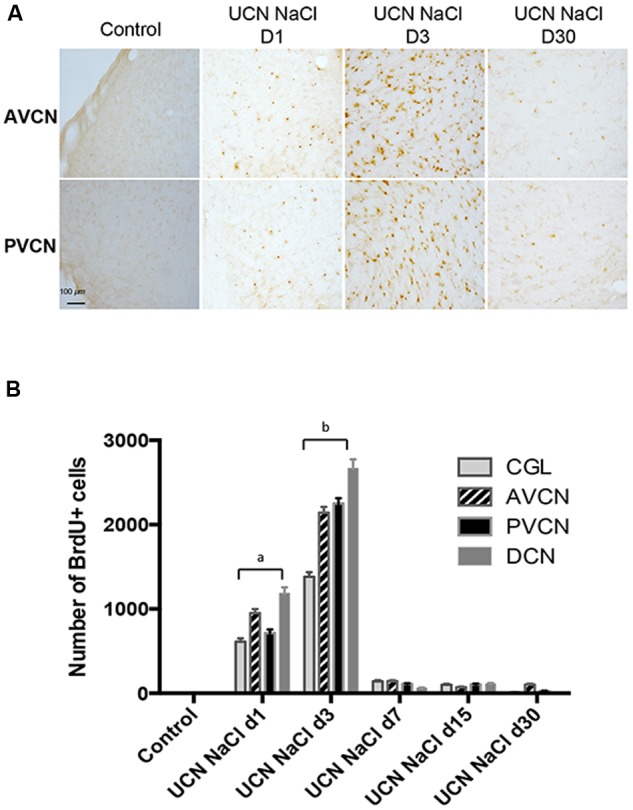
**Reactive cell proliferation occurs after unilateral cochlear neurectomy (UCN) in a time-sensitive manner.** **(A)** Photomicrographs showing 5-bromo-2′-deoxyuridine (BrdU) immunostainings in the deafferented cat AVCN and PVCN submitted to UCN and analyzed at post-lesion days 1, 3, and 30 (D1, D3, and D30, respectively). Animals received a BrdU injection (i.p., 200 mg/kg body weight) 3 h before sacrifice. Scale bar: 100 μm for lower magnification. **(B)** Quantitative evaluation. Histograms comparing the mean values (±Standard Error of the Mean, SEM) of the number of BrdU-immunopositive cells in the deafferented CN at D1, D3, and D30 after UCN. Different letters indicate significant differences between groups of animals and assessed by ANOVA followed by the Scheffé test, *p* < 0.0001, see **Table 1**. a: significantly different from control, UCN d3, UCN d7, UCN d15, and UCN d30 groups. b: significantly different from control, UCN d1, UCN d7, UCN d15, and UCN d30 groups. Only values recorded on the lesioned side are illustrated. *n* = 4 animals per group.

In sham-operated cats, no BrdU-Ir nuclei were detected in the CN. The regions of interest and the lesion-induced changes in BrdU-Ir within the CN are illustrated in **Figures 2A,B**. In the UCN group of cats, BrdU-Ir cells were exclusively restricted to the deafferented CN. The onset of cell proliferation began 1 day after the cochlear nerve section, peaked at 3 days and then decreased to reach control values at 30 days post-UCN. The quantitative analysis showed a significantly increased number of BrdU-Ir nuclei by the first day and peaked at 3 days after UCN for the four main CN sub-divisions (+1382% in the CGL, +2140.93% in the AVCN, +2252.07% in the PVCN, and +2667.03% in the DCN compared to controls, *P* < 0.0001). Repeated measures analysis of variance of the data are given in **Table 1**. They indicate that the number of BrdU immunoreactive cells differed significantly with regards to group (*P* < 0.0001), side (*P* < 0.0001), and CN (*P* < 0.0001). The interaction between these three factors (group, side, and CN) was also highly significant (*P* < 0.0001) (**Table 1**). Treatment with Muscimol significantly enhanced the number of BrdU-Ir cells at day 3 post-lesion in all subdivisions of cochlear nuclei compared with the UCN-NaCl group (+125% in the CGL, +125% in the AVCN, +117% in the PVCN, and +112% in the DCN compared to UCN-NaCl group, *P* < 0.0001).

**Table 1:** Statistical analysis of the effects of unilateral cochlear neurectomy (UCN) on the time course of cell proliferation in the cochlear nuclei complex of adult cats.

Source of variation	df	*F*	*P*
**Number of BrdU positive cells**
Group	5	7702.02	0.0001^∗^
Side	1	19064.15	0.0001^∗^
Group × side	5	7702.02	0.0001^∗^
Cochlear nucleus	3	248.74	0.0001^∗^
Group × Cochlear nucleus	15	146.74	0.0001^∗^
Side × Cochlear nucleus	3	248.74	0.0001^∗^
Group × side × Cochlear nucleus	15	146.74	0.0001^∗^


*Repeated measures analysis of variance of the changes in the BrdU-immunoreactive cells after UCN. The main fixed effects providing the source of variation among animals were group (that is, sham versus unilateral- cochlear -lesioned cats as a function of the survival postoperative period) and side (intact versus deafferented), and cochlear nucleus (AVCN: anteroventral cochlear nucleus; PVCN: posteroventral cochlear nucleus; CDN: dorsal cochlear nucleus and CGL: cochlear granular cell layer). Degree of freedom (d.f.), Scheffe test (F), and probability level (p) are reported. ^∗^Indicates significant variations between variables and/or their interactions.*

**FIGURE 3:**
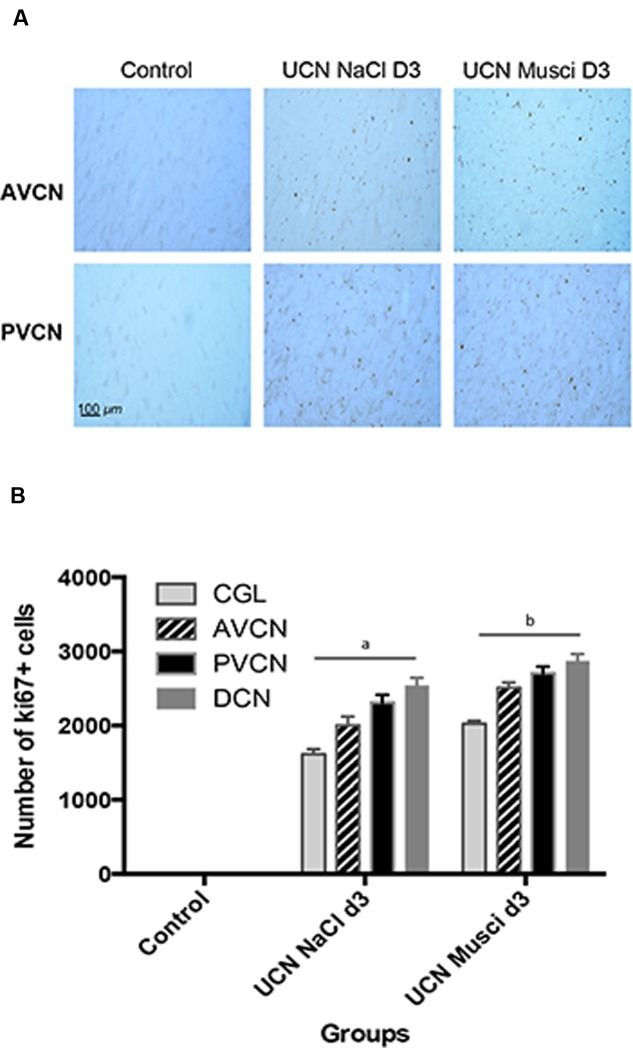
**Ki67 immunostaining was used to confirm BrdU cell proliferation.** **(A)** Photomicrographs showing Ki67 immunostainings in the deafferented AVCN and PVCN of control cats and cats infused with NaCl or Muscimol immediately after UCN and at sacrifice (D3). Scale bar: 100 μm. **(B)** Quantitative evaluation. Histograms comparing the mean values (±SEM) of Ki67-immunopositive cells in the deafferented CN 3 days after UCN in control cats or cats submitted to NaCl or Muscimol infusion using ANOVA followed by the Scheffé test (*p* < 0.0001, see **Table 2**). Data from both sides of control animals were pooled for direct comparison with the subgroups of lesioned cats. Different letters indicate significant differences between groups of animals in each CN: a: significantly different from control and UCN Muscimol groups; b: significantly different from control and UCN NaCl groups. Only values recorded on the lesioned side are illustrated. *n* = 4 animals per group.

The time course of Ki67 immunoreactivity was similar to that observed for BrdU immunoreactivity (**Figure 3** and **Table 2**). Ki67 marker was used in addition to BrdU to confirm that BrdU had been incorporated into mitotic cells and did not correspond to dying cells or a DNA repair mechanism (Dutheil et al., 2009). In sham-operated cats, no Ki67 nuclei were detected in the CN. A strong and significant number of Ki67 immunoreactive cells were observed 3 days after cochlear nerve section in the whole CN (+10616% in the CGL, +2006% in the AVCN, +2310% in the PVCN, and +2536% in the DCN compared to controls, *P* < 0.0001). In contrast, 3 days after UCN, AraC blocked the cell proliferation in the whole CN. The number of ki67-Ir nuclei in the CN was close to zero in cats infused with the antimitotic drug immediately after UCN (UCN-AraC D3 group), thus confirming the efficacy of AraC in blocking cell proliferation. The same result was previously described in deafferented vestibular nuclei after unilateral vestibular neurectomy and AraC infusion in the adult cat (Dutheil et al., 2009). Repeated measures analysis of variance of the data are given in **Table 2**. They indicate that the number of Ki67 immunoreactive cells differed significantly with regards to group (*P* < 0.0001), side (*P* < 0.0001), and CN (*P* < 0.0001). The interaction between these three factors (group, side, and CN) was also highly significant (*P* < 0.0001). There were no Ki67-immunoreactive cells in any of the subdivisions of the CN contralateral to the lesion.

**Table 2:** Statistical analysis of the effects of UCN on the cell proliferation in the cochlear nuclei complex of adult cats.

Source of variation	df	*F*	*P*
**Number of Ki positive cells**
Group	2	5293.69	0.0001^∗^
Cochlear nucleus	3	137.64	0.0001^∗^
Group × Cochlear nucleus	6	35.39	0.0001^∗^
Side	1	20692.91	0.0001^∗^
Group × side	2	5293.69	0.0001^∗^
Cochlear nucleus × side	3	137.64	0.0001^∗^
Group × side × Cochlear nucleus	6	35.39	0.0001^∗^


*Repeated measures analysis of variance of the changes in the Ki67-immunoreactive cells after UCN. The main fixed effects providing the source of variation among animals were group (that is, control cats or unilateral cochlear neurectomized cats infused with NaCl or Muscimol durind 3 days), side (intact versus deafferented), and cochlear nucleus (AVCN: anteroventral cochlear nucleus; PVCN: posteroventral cochlear nucleus; CDN: dorsal cochlear nucleus and CGL: cochlear granular cell layer). Degree of freedom (d.f.), Scheffe test (F), and probability level (p) are reported. ^∗^Indicates significant variations between variables and/or their interactions.*

### Survival of BrdU Immunoreactive Cells

**FIGURE 4:**
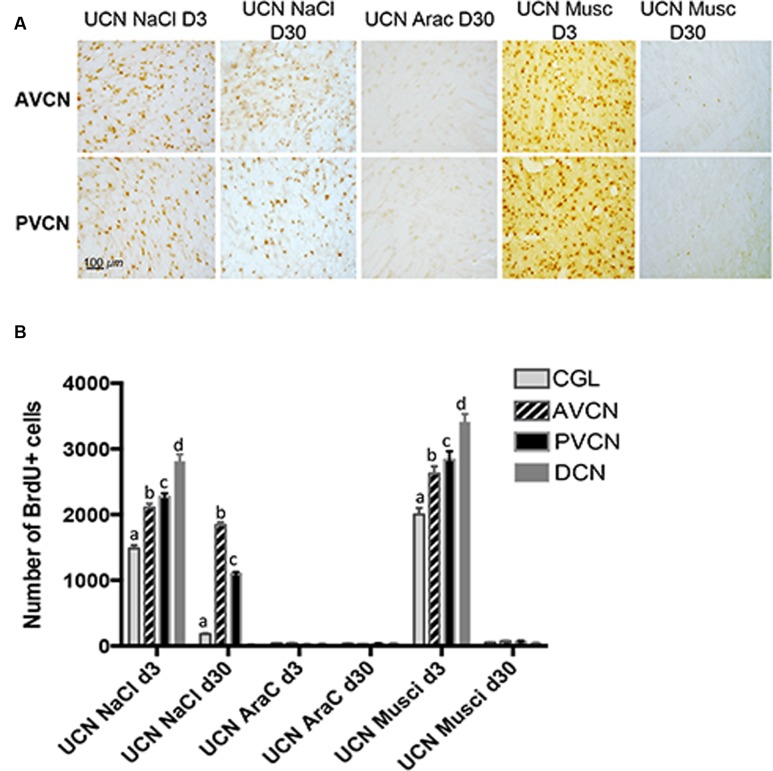
**Newborn cells generated 3 days after UCN survived up to 1 month depending on the treatment administrated.** **(A)** Illustrations of BrdU immunoreactivity in both the deafferented AVCN and PVCN at different time points (post-lesion D3 or D30) in UCN animals treated with NaCl-, AraC-, or Muscimol-intra-cerebroventricular infusion. Note that in AraC treated animals, no BrdU+ cells were observed at D3 (data not shown) or D30. Scale bar: 100 μm and *n* = 4 animals per group. **(B)** Histograms showing the effects of drug infusion (NaCl, AraC, or Muscimol) on the number of BrdU-immunopositive cells observed in the deafferented cochlear nuclei. Only values recorded on the lesioned side are illustrated. Different letters indicate significant differences between all other groups of animals. Analyzes were assessed by ANOVA followed by the Scheffé test for all VN and groups (*p* < 0.0001, see **Table 3**).

To study the survival of the newly generated cells stained with BrdU at 3 days (peak of cell proliferation), the sub-groups of animals were killed 27 days after BrdU injection. We found that ∼60% of BrdU^+^ cells survived in the UCN-NaCL group. These data indicate a loss of BrdU-Ir nuclei in the CN of cats injected at 3 days and killed 30 days after UCN compared to the group of cats injected at the same post-lesion delay and killed 3 h later. This reduction suggests that some of the cells that incorporated BrdU died. **Figure 4** shows the mean numbers of BrdU-Ir nuclei that survived in the subdivisions of CN in the different groups of cats. In the UCN-NaCl group, survival ratio was highest in the AVCN (87.3%) than in the other CN subdivisions (48.3 and 12.2% in the PVCN and the CGL, respectively) (**Table 3**). The survival ratio was very low (<2%) in the DCN (UCN-NaCl group) and in all CN subdivisions in animals treated with muscimol (UCN-muscimol group) and AraC (UCN-AraC group). Repeated measures analysis of variance of the data are given in **Table 3**. They indicate that the number of surviving BrdU immunoreactive cells differed significantly with regard to group (*P* < 0.0001), side (*P* < 0.0001), and CN (*P* < 0.0001). The interaction between these three factors (group, side, and CN) was also highly significant (*P* < 0.0001).

**Table 3:** Statistical analysis of the survival of newly generated cells by comparing cats receiving BrdU injection 3 days after UCN and perfused 3 h after (protocol 1) and those receiving BrdU at the same time but perfused 27 days after (protocol 2).

Source of variation	df	*F*	*P*
**Number of BrdU surviving cells**
Group	2	6360.35	0.0001^∗^
Cochlear nucleus	3	369.20	0.0001^∗^
Group × Cochlear nucleus	6	205.55	0.0001^∗^
Postoperative time	1	13207.51	0.0001^∗^
Group × Postoperative time	2	4296.23	0.0001^∗^
Cochlear nucleus × Postoperative time	3	435.34	0.0001^∗^
Group × Cochlear nucleus × Postoperative time	6	214.46	0.0001^∗^


*Repeated measures analysis of variance of the changes in the BrdU-immunoreactive cells after UCN. The main fixed effects providing the source of variation among animals were group (unilateral cochlear neurectomized cats infused with NaCl, Muscimol, or AraC), the survival postoperative (period 3 days versus 30 days), side (intact versus deafferented) and cochlear nucleus (AVCN: anteroventral cochlear nucleus; PVCN: posteroventral cochlear nucleus; CDN: dorsal cochlear nucleus and CGL: cochlear granular cell layer). Degree of freedom (d.f.), Scheffe test (F), and probability level (p) are reported. ^∗^Indicates the significant variations between variables and/or their interactions.*

### Phenotype of Newly Generated Cells

**FIGURE 5:**
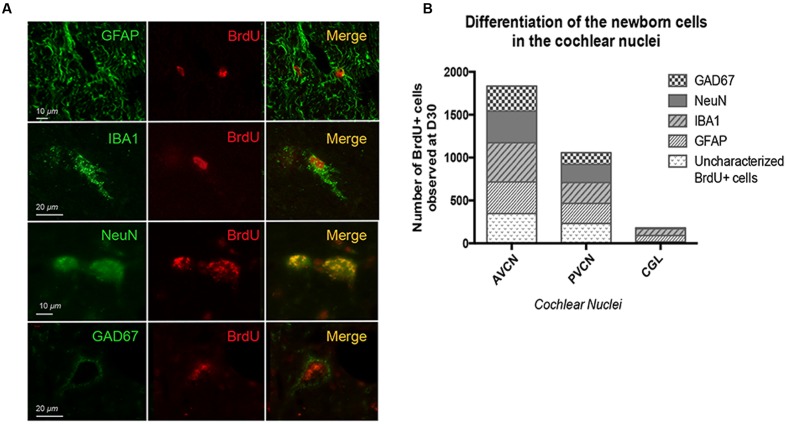
**Thirty days after UCN, glial fibrillary acidic protein (GFAP) and glutamic acid decarboxylase (GAD67) immunoreactivity are differentially expressed in the cochlear nuclei of cats submitted to a cochlear neurectomy.** **(A)** Double immunostaining confocal analysis of differentiated newly generated cells processed on consecutive serial sections. The BrdU+ nuclei are in red and the other markers of differentiation: GFAP+, IBA1+, NeuN+, and GAD 67+ in green. Illustrations were made in the deafferented AVCN nucleus of cats 30 days after cochlear neurectomy. **(B)** Histograms illustrating the percentage of GFAP, IBA1, NeuN, and GAD67 positive cells among the BrdU positive cells observed in the different sub-divisions of CN.

Cell differentiation was investigated by double immuno-histochemical labeling using BrdU combined with the four specific cell type markers: GFAP, NeuN, IBA1, and GAD67 (see Materials and Methods). The double labeling has been quantified only at 30 days in the UCN-NaCl group since co-labeled cells were not observed at the day 3 and no BrdU immunoreactive cells survived in the UCN-AraC and UCN-muscimol groups. The photomicrographs in **Figure 5A** show the colocalization of BrdU^+^ with GFAP^+^, IBA1^+^ NeuN^+^, and GAD 67^+^ cells observed in the deafferented AVCN at 30 days post-lesion in the UCN-NaCl group. The fate of the newly generated cells varied depending on the CN sub-division (**Figure 5B**). The results are expressed in percent defined as the ratio between the mean number of immunopositive-elements colocalizing a cell type marker (GFAP, IBA1, NeuN, or GAD 67) and BrdU relative to the total mean number of BrdU^+^ nuclei counted in the areas of quantification. For the glial (GFAP and IBA1) and neuronal marker (NeuN), the newly generated cells differentiated approximately in similar proportion in the AVCN (20, 25, and 20%) and the PVCN (22, 23, and 20%). In the CGL, by contrast, GFAP and IBA1 labeling were higher (40 and 45%) than NeuN labeling (5%). The mean number of newly generated GAD67^+^ neurons varied depending on the CN subdivision (**Figure 5B**): 16, 13, and 0% in the AVCN, PVCN, and CGL, respectively. A non-negligible percentage of cells with an undetermined phenotype was observed in all the CN subdivisions (19% in the CVA, 22% in the CVP, and 10% in the CGL).

### KCC2

**FIGURE 6:**
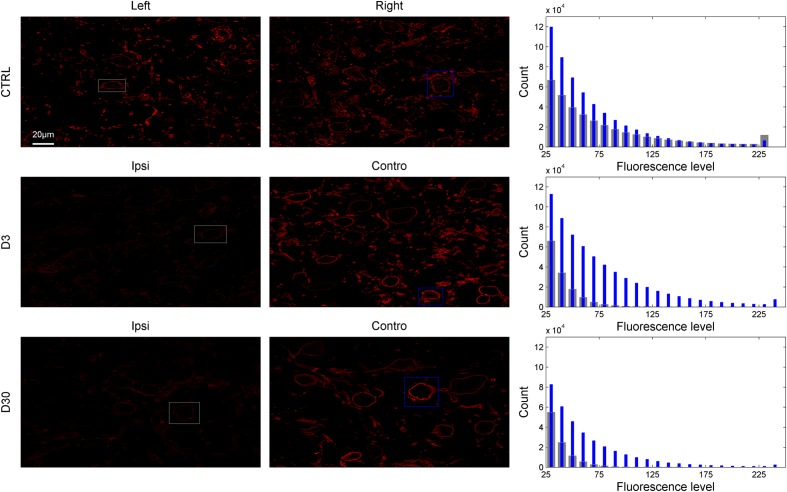
**Examples of entire slices showing KCC2-related fluorescence in control and lesioned animals (D3 and D30 post-lesion) in the left and right VCN (or VCN ipsilateral and contralateral side to the lesion; first and second column).** The corresponding distribution of fluorescence from the slices is shown in the third column (gray bars: left or ipsilateral to the lesion, blue bars: right or contralateral to the lesion). The region of interests delineated by gray and blue rectangles in the first and second columns are showed in **Figure 7** at higher magnification.

**FIGURE 7:**
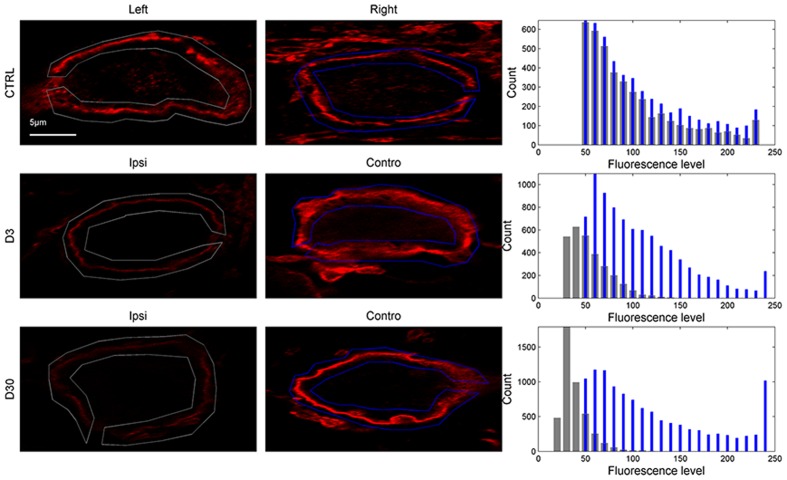
**KCC2-related fluorescence of cells bodies in control and lesioned animals from the region of interests delineated in **Figure 6** (first and second column).** The distribution of fluorescence levels from the region of interest drawn around the plasma membrane is shown in the third column (gray bars: left or ipsilateral to the lesion, blue bars: right or contralateral to the lesion).

Simple visual inspection of examples shown in **Figures 6** and **7** suggests that KCC2 expression is dramatically downregulated in the ventral CN ipsilateral to the lesion at 3 and 30 days post-lesion. The regions of interest delineated by the rectangles in **Figure 6** are shown in **Figure 7** at a larger magnification, allowing for a better view of the cell bodies and membrane fluorescence. Contours (gray: ipsilateral to the lesion, and blue: contralateral to the lesion) drawn around the cell body membrane define the region of interest from which fluorescence level is derived. At this magnification, it is again very clear that KCC2 density at the plasma membrane is dramatically downregulated in the cochlear nuclei ipsilateral to the lesion. The distribution of membrane fluorescence is shown in the right column of **Figure 7**.

**FIGURE 8:**
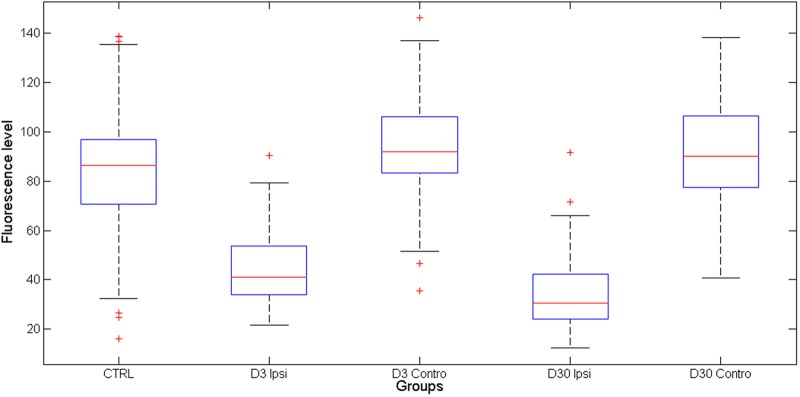
**Averaged KCC2-related fluorescence in control and lesioned (in CN ipsilateral and contralateral to the lesion) animals.** KCC2 is strongly downregulated on the VCN ipsilateral to the lesion.

The averaged KCC2 fluorescence level for each condition (control animals: *n* = 110 cells; lesioned animals, ipsilateral to the lesion, day 3 *n* = 101 cells; lesioned animals, contralateral to the lesion, day 3 *n* = 75 cells; lesioned animals, ipsilateral to the lesion, day 30 *n* = 97 cells; lesioned animals, contralateral to the lesion, day 30 *n* = 85 cells) is shown in **Figure 8**. In lesioned animals, the Mann–Whitney test revealed that KCC2-related fluorescence in the ventral CN ipsilateral to the lesion is significantly lower than fluorescence in ventral CN contralateral to the lesion at days 3 and 30 post-lesion (*p* < 0.05). The level of KCC2-related fluorescence was also significantly reduced in the CN ipsilateral to the lesion when compared to the CN in control animals (*p* < 0.05).

## Discussion

### Cochlear Lesion-Induced Mitotic Activity in the Deafferented Cochlear Nuclei

The present study shows an intense BrdU immunolabeling after unilateral section of the cochlear nerve in the adult cat that is restricted exclusively to the cochlear nuclei ipsilateral to the lesion. The presence of Ki67-Ir cells in the CN after UCN strongly suggests that BrdU immunolabeling is related to mitotic activity instead of DNA repair or apoptotic events. Cell proliferation was observed as early as 1 day post-lesion, with a peak at 3 days, a reduction at 7 days, and a total lack of BrdU-Ir cells at 30 days. At 1 month post-lesion, many of the BrdU-Ir cells survived and differentiated giving rise to microglia, astrocytes and neurons in all CN subdivisions (except DCN). A substantial part of the newborn neurons acquired a GABAergic phenotype, especially in the AVCN.

Our study corroborates an earlier study that reported cell proliferation, survival, and differentiation of the newborn cells in the CN of rats after bilateral cochlear lesions (Zheng et al., 2011). The two studies differ, however, on several aspects. The peak of cell proliferation is delayed in our study (day 3 post-lesion) compared to that of Zheng’s study (day 2 post-lesion). Moreover, the number of newborn cells observed at the peak of cell proliferation was much higher in our study compared to that reported in Zheng’s study. Concerning the survival ratio, the two studies cannot be compared as, unlike in Zheng’s study, our study analyzed the different sub-divisions of CN separately. The type of cochlear lesion can in part account for the discrepancies between these two studies. Indeed, the cochleas are bilaterally destroyed in Zheng’s study, which largely spares the cochlear nerve. In our study, on the other hand, the cochlear nerve is sectioned unilaterally. Nerve section is known to produce dramatic effects on the cellular microenvironment in the innervated tissue, including Wallerian degeneration and strong inflammatory processes (Liberge et al., 2010; Gaudet et al., 2011). This particular biochemical environment triggered by cochlear nerve section may have promoted reactive cell proliferation in the CN.

Cell proliferation, survival, and differentiation have also been observed in the vestibular nuclei after unilateral vestibular nerve section, but not after unilateral intratympanic injection of tetrodotoxin or labyrinthectomy (Tighilet et al., 2007; Dutheil et al., 2009, 2011). Labyrinthectomy in the vestibular system can be considered equivalent to cochlear destruction in the auditory system, i.e., the sensory organs are destroyed but the vestibular nerve is left intact. These results (cell proliferation and differentiation after cochlear destruction but not after labyrinthectomy) may indicate a proneness of the auditory system to reactive neurogenesis over the vestibular system. A putatively stronger inflammatory reaction in the central auditory system to a peripheral lesion may account for the differences observed between the two sensory systems (Baizer et al., 2015). Recently, cell proliferation has been reported in the cochlear nuclei after unilateral noise trauma (Zheng et al., 2015). This result is consistent with the idea that even moderate cochlear lesions may trigger central modifications that are favorable to cell proliferation.

Compared to the neurogenesis occurring spontaneously, the high percentage of cell proliferation and survival ratio reported in this and other studies can be surprising. However, the two types of cell proliferation (spontaneous and reactive) are very different. Indeed, the adult mammalian brain is considered mostly as non-neurogenic, except in the subventricular zone of the lateral ventricles and the subgranular zone of the dentate gyrus. It is only in pathological conditions or after severe injury that anti-neurogenic influences can be removed and that cell proliferation can occur in usually non-neurogenic regions. It is likely that the two types of cell proliferation are governed by different mechanisms. *In fine*, this could explain the high level of cell proliferation and survival observed in the deafferented cochlear nuclei after unilateral cochlear nerve section.

Regarding the origin of the reactive proliferating cells in the cochlear nuclei several hypotheses can be proposed. Either they result from a local origin, i.e., the intraparenchymental neural precursors in the cochlear nuclei (Manohar et al., 2012) or they migrate from other brain structures. In the present study, we observed cell proliferation as early as 1 day post-lesion, with a peak at 3 days. The precociousness of the cell proliferation suggests a local origin of the precursors.

While many of the newborn cells survive in the ventral CN 30 days after the cochlear nerve section, there were no surviving cells in the DCN. Moreover, the treatment with the GABA_A_ agonist muscimol (starting with UCN and continued until sacrifice) does not prevent cell proliferation but inhibits completely survival. These results raise questions concerning the mechanisms that facilitate/prevent neuronal survival after cell proliferation. It has been proposed that the survival of newborn neurons is activity-dependent, requiring normal activation of NMDA receptors and the sub-sequent influx of calcium into the cells (Tashiro et al., 2006). However, the NMDA receptor activity should not be abnormally strong as too much intracellular calcium can cause cell death (Hardingham and Bading, 2003). The origin of intra-cellular calcium, whether it comes from intra- or extra-synaptic NMDA receptor activity, may also be important for the fate of neural cells (Hardingham and Bading, 2003). The absence of survival in the dorsal CN after cochlear nerve section may result from the relatively preserved (and maybe too large) neural activity in this nucleus. This is possibly due to non-auditory inputs (Shore and Zhou, 2006), even after cochlear destruction (Koerber et al., 1966). In the same vein, muscimol could have increased neural activity (see below the functional implications of KCC2 down-regulation) thereby leading to an excessive increase of intracellular calcium concentration and death of newborn neurons (Furukawa et al., 2000).

### Deafferentation Induced a Down-Regulation of KCC2 Co-Transporters in the Ventral CN

For the first time in the auditory modality, the present study shows that KCC2 co-transporters are dramatically downregulated (by 50% on average) in the cochlear nuclei after unilateral deafferentation. This reduction in KCC2 density was observed on the membrane of the cell body as early as 3 days post-lesion and did not recover by 30 days post-lesion. Our results are in strong agreement with other studies showing a down-regulation of KCC2 after stroke or other neural injuries at different locations (spinal chord, sciatic, and vagal nerve) (Nabekura et al., 2002; Coull et al., 2003; Boulenguez et al., 2010; Jaenisch et al., 2010). In auditory modality, only one study investigated the putative changes of KCC2 in auditory centers after hearing loss. It reported that KCC2 gene expression was unchanged after a bilateral cochlear ablation (Vale et al., 2003). Taken together, these results suggest that hearing loss can downregulate KCC2 in the plasma membrane of central neurons without changing the global expression level of these proteins.

Much effort has been devoted to understanding the regulatory mechanisms of KCC2 activity including: their transcriptional levels, their density in the neuronal plasma membrane and the fine-tuning of their intrinsic transport properties. It is known that KCC2 activity is regulated, at least in part, by phosphorylation/dephosphorylation mechanisms (Kahle and Delpire, 2016). The WNK-SPAK/OSR1 kinase complex has been suggested to play an important regulatory role on cation–chloride cotransporters. Indeed, WNKs stimulate the kinases SPAK and OSR1, which directly phosphorylate and inhibit KCC2, while stimulating sodium-driven cation–chloride cotransporters (NKCC, which enhances intra-cellular chloride level) (Alessi et al., 2014). On the contrary, the dephosphorylation of KCC2 at Thr906/Th1007 stimulates KCC2 activity by preventing the phosphorylation of WNK1-SPAK at these sites (Rinehart et al., 2009). Moreover, the phosphorylation of KCC2 at another site (Ser940) by protein kinase C has been shown to raise the membrane expression and intrinsic transport rate of KCC2 (Lee et al., 2007; Chamma et al., 2013).

The reduction of the sensory inputs and/or the modifications of the cellular micro-environment produced by unilateral nerve section have somehow to be sensed by neurons triggering a molecular cascade leading to dramatic reduction of KCC2 levels. The brain-derived neurotrophic factor (BDNF) has been suggested as playing a role in the activity-dependent regulation of KCC2 through neuronal TrKB receptors (Rivera et al., 2004). Indeed, the BDNF released by activated microglia has been shown to downregulate KCC2 (Coull et al., 2005; Ferrini and De Koninck, 2013). Microglia, activated by various molecules such as cytokine (Tsuda et al., 2009), up-regulates the expression of the purinergic receptor P2X4R, which is required for synthesizing and releasing BDNF (Ferrini and De Koninck, 2013). The expression of BDNF is also enhanced during chronic stress (Lippmann et al., 2007). In our unilateral vestibulocochlear neurectomy model, we demonstrated a long-lasting activation of the hypothalamo–pituitary–adrenal axis and an up-regulation of BDNF and its TrKB receptor in the deafferented vestibular nuclei (Tighilet et al., 2009). Similar results have been observed in the CN (data not shown). After unilateral vestibular neurectomy, we showed recently that intracerebroventricular infusion of BDNF increases dramatically cell proliferation and survival in the vestibular nuclei, whereas the antagonist of TrKB receptor K252a produced the reverse. Interestingly, K252a has been shown to upregulate the KCC2 co-transporters (Dutheil et al., 2016). Further studies are needed to investigate whether BDNF has the same effects in the cochlear nuclei after cochlear nerve section.

### Functional Implications of Cell Proliferation and KCC2 Down-Regulation

Previous work in our laboratory described the occurrence of reactive neurogenesis after unilateral vestibular nerve section (Tighilet et al., 2007; Dutheil et al., 2009, 2011). Interestingly, this reactive neurogenesis played a critical role in restoring the vestibular function (Dutheil et al., 2009, 2011). This result suggests that newborn neurons can be integrated in a functional neural network and play an adaptive role in the vestibular recovery after peripheral damage. Newborn neurons could have contributed to restore a normal neuronal activity on the lesioned side (Zennou-Azogui et al., 1993; Ris et al., 1995). In the auditory modality, many studies showed that cochlear lesions are followed by neural hyperactivity in the auditory centers (Kaltenbach et al., 2000; Noreña and Eggermont, 2003; Noreña et al., 2003; Cai et al., 2009; Mulders and Robertson, 2009; Kalappa et al., 2014). For example, neural activity estimated from 2-deoxyglucose was reduced 2 h post cochlear nerve section but was restored (and even potentially enhanced) a few weeks after the lesion (Sasaki et al., 1980). In conclusion, this study and others suggest that reactive neurogenesis may be part of the homeostatic mechanism repertoire rapidly triggered after sensory deafferentation to restore a normal averaged activity in sensory centers.

In the general context of the many resources and energy deployed by the auditory system to restore neural activity after deafferentation (Noreña, 2011), the differentiation of a substantial amount of newborn neurons into the GABAergic phenotype (i.e., inhibitory) can appear contradictory. However, this contradiction is only apparent. Indeed, the action of GABA on the membrane potential depends on the intra-cellular concentration of chloride ion which is maintained at low levels in adults by KCC2 co-transporters. By showing that KCC2 co-transporters are strongly downregulated after cochlear deafferentation, the present study suggests that the polarity of GABA on membrane potential became depolarizing (Coull et al., 2003; Boulenguez et al., 2010). *In fine*, this result implies that GABAergic neurons may contribute to restore normal neural activity after sensory deafferentation.

Finally, while the down-regulation of KCC2 in adults after nerve injury, ischemia, inflammation, or deafferentation can be adaptive in most cases, it may sometimes come at a price, i.e., it can be accompanied by abnormal neural activity resulting in various diseases, namely autism, neuropathic pain, spasticity, and epilepsy (Coull et al., 2003; Boulenguez et al., 2010; Jaenisch et al., 2010; Toda et al., 2014; Sivakumaran et al., 2015). In the auditory modality, tinnitus and hyperacusis have been suggested to be a by-product of homeostatic mechanisms triggered by hearing loss (Schaette and Kempter, 2006; Noreña, 2011). Tinnitus is largely prevalent in the general population and even more so in subjects with profound hearing loss, including subjects who underwent cochlear nerve section surgery (Quaranta et al., 2004; Baguley et al., 2006; Baguley and Atlas, 2007; McCormack et al., 2014). Regarding the possible contribution of KCC2 down-regulation in some neurological diseases, including neuropathic pain [which is often compared to tinnitus – (Møller, 2007)], and the dramatic decrease of KCC2 after cochlear nerve section (this study), it is tempting to propose that down-regulation of KCC2 after hearing loss may play a role in the generation of tinnitus and hyperacusis.

### Clinical Relevance

The present study shows that KCC2 is downregulated after hearing loss suggesting that GABA can become excitatory. This result is very important as it implies that GABA agonists are not necessarily adapted to treat neurological disorders associated to neural hyperactivity, including tinnitus and hyperacusis. Moreover, the putative down-regulation of KCC2 after hearing loss may account for the mixed effects of GABA agonist on tinnitus (Johnson et al., 1993; Jalali et al., 2009; Aazh et al., 2011). In this context, KCC2 may represent a much more promising pharmacotherapeutic target for treating tinnitus and hyperacusis. This approach is being developed for treating neurological diseases such as chronic pain (Doyon et al., 2013; Gagnon et al., 2013; Kahle and Delpire, 2016).

Also, it is likely that the inflammatory processes in the cochlear nuclei triggered by cochlear nerve section contribute to the neural changes observed in this study. One can speculate that reducing central inflammation, and/or limiting cell proliferation and neural differentiation triggered by sensory deafferentation, may prevent the emergence of abnormal activity, and tinnitus and hyperacusis. A recent study from our group reported that an antagonist of BDNF can reduce cell proliferation and survival and up-regulate KC22 co-transporters (Dutheil et al., 2016). This suggests that antagonizing the BDNF signaling pathway after cochlear lesion may counteract the mechanisms resulting in central hyperactivity and potentially those involved in the generation of tinnitus and hyperacusis.

## Conclusion

The present study demonstrates cell proliferation and differentiation in the cochlear nuclei after cochlear nerve section. It also shows a dramatic down-regulation of KCC2 in the CN after sensory deafferentation, suggesting that GABA became excitatory. These two mechanisms add to the vast repertoire of neural mechanisms triggered by hearing loss thought to be homeostatic regarding neural activity. However, these mechanisms may come at a price: they may be involved in the generation of tinnitus and hyperacusis.

## Author Contributions

BT designed, made experiments, analyzed data, and wrote paper. AN designed, analyzed data, and wrote paper. SD made experiments and analyzed data. MS analyzed data and wrote paper.

## Conflict of Interest Statement

The authors declare that the research was conducted in the absence of any commercial or financial relationships that could be construed as a potential conflict of interest.
